# Correlation between lung cancer probability and number of pulmonary nodules in baseline computed tomography lung cancer screening: A retrospective study based on the Chinese population

**DOI:** 10.3389/fonc.2022.1061242

**Published:** 2023-01-04

**Authors:** Quanyang Wu, Shijun Zhao, Yao Huang, Jianwei Wang, Wei Tang, Lina Zhou, Linlin Qi, Zewei Zhang, Yuting Xie, Jiaxing Zhang, Hongjia Li, Ning Wu

**Affiliations:** ^1^ Department of Diagnostic Radiology, National Cancer Center/National Clinical Research Center for Cancer/Cancer Hospital, Chinese Academy of Medical Sciences and Peking Union Medical College, Beijing, China; ^2^ PET-CT Center, National Cancer Center/National Clinical Research Center for Cancer/Cancer Hospital, Chinese Academy of Medical Sciences and Peking Union Medical College, Beijing, China; ^3^ Department of Cancer Epidemiology, National Cancer Center/National Clinical Research Center for Cancer/Cancer Hospital, Chinese Academy of Medical Sciences and Peking Union Medical College, Beijing, China; ^4^ Department of Diagnostic Radiology, National Cancer Center/National Clinical Research Center for Cancer/Hebei Cancer Hospital, Chinese Academy of Medical Sciences, Langfang, China

**Keywords:** lung cancer probability, low-dose computed tomography, pulmonary nodules, nodule numbers, screening

## Abstract

**Background:**

Screening for lung cancer with LDCT detects a large number of nodules. However, it is unclear whether nodule number influences lung cancer probability. This study aimed to acquire deeply insight into the distribution characteristics of nodule number in the Chinese population and to reveal the association between the nodule number and the probability of lung cancer (LC).

**Methods:**

10,167 asymptomatic participants who underwent LDCT LC screening were collected. Noncalcified nodules larger than 4 mm were included. The nodule number per participant was determined. We defined five categories according to the number of nodules (based on nodule type and size): one, two, three, four, and more than four nodules. We stratified the nodules as groups A, B, and C and participants as Amax, Bmax, and Cmax groups, and explored the association between nodule number and the probability of LC on nodule and participant levels.

**Results:**

97 participants were confirmed to have LC. The probabilities of LC were 49/1719, 22/689, 11/327, 6/166, and 9/175 in participants with one, two, three, four, and more than four nodules (p>0.05), respectively. In the Bmax group, the probability of LC was significantly higher in participants with one nodule than those with >4 nodules (p<0.05), and the probability of LC showed a negative linear trend with increasing nodule numbers (p<0.05). Based on the nodule-level analyses, in Group B, LC probability was significantly higher when participants had a solitary nodule than when they had >4 nodules (p<0.05).

**Conclusion:**

LC probability does not significantly change with the number of nodules. However, when stratified by the nodule size, the effect of nodule number on LC probability was nodule-size dependent, and greater attention and active follow-up are required for solitary nodules especially SNs/solid component of PSNs measuring 6-15 mm or NSNs measuring 8-15 mm. Assessing the nodule number in conjunction with nodule size in baseline LDCT LC screening is considered beneficial.

## Introduction

According to GLOBOCAN 2020, lung cancer (LC) ranks second in the world’s newly occurring malignant tumors and remains the leading cause of cancer-related mortality worldwide (1). Most patients with LC are already in the moderate to late stages at diagnosis (2, 3), and their 5-year survival rate are only 21% (4). Therefore, performing LC screening to improve the rate of early diagnosis and treatment is of great significance to improve patient survival and reduce LC mortality. Data from a nonrandomized controlled study of the International Early Lung Cancer Action Program (I-ELCAP) indicate that LC can be detected at an early stage during low-dose computed tomography (LDCT) screening, with stage I LC numbers more than 80% of cases. With timely treatment, the expected 10-year survival rate is as high as 88% (5). The results of the National Lung Screening Trial (NLST) in the United States and the Dutch-Belgian Randomized Lung Cancer Screening Trial (NELSON) in Europe showed that LDCT screening can reduce LC mortality (6, 7). The characteristics of LC screening in Asian populations differ from those in Europe and America, especially the proportion of non-smokers in female lung cancer participants is significantly higher than that in Europe and America (8). The results of the NLST showed that 26.8% of the participants had pulmonary nodules at the 4mm threshold criterion and pulmonary nodules are clinically significant because they may be the first presentation of LC (9). The main concern of LC screening is the early identification of their differentiation between benign and malignant in indeterminate pulmonary nodules. Some nodule characteristics, such as nodule diameter, speculation, location of the superior lobe, and the solid components, may be associated with an increased probability of LC (10, 11). Volume and mass also has been reported to potentially reflect the natural growth history of nodules (12, 13). However, nodule size is still the first consideration in LC risk assessment.

Malignancy estimation and management recommendations are primarily based on expert consensus or data from research trials, and nodule-grade classification is often based on nodule size. Risk assessment of participants with multiple nodules is often based on the highest risk nodule, usually the largest nodule, but smaller nodules should not be ignored. However, one of the neglected aspects in real-world clinical implementation is the nodule number detected in patients during LDCT LC screening. Although multiple nodules are often identified during LDCT screening, the number of nodules is frequently ignored in the risk assessment of LC. A limited analysis of multinodularity and LC risk was conducted for nodules detected in NELSON trial. Nodule number was found to be ambiguous in relation to LC probability in participants, with LC probability varying with the number of nodules (14). The effect of the number of nodules on the probability of LC deserves further study. This study aimed to explore the correlation between the probability of LC and number of nodules detected during baseline LDCT LC screening within the Chinese population.

## Materials and methods

### Study population

LDCT screening data were collected and analyzed for 10167 asymptomatic participants undergoing early LC screening at the National Cancer Center from 2010 to 2018. The opportunistic LDCT LC screening inclusion criteria were as follows: (a) aged 40–80 years; (b) participants with no clinical symptoms; (c) participants with no cancer history within 5 years (via registry); and (d) participants with good general physical condition and willingness to undergo invasive diagnostic assessment and treatment, such as biopsy, puncture, and surgery, required for positive screening results. Participants who met the basic eligibility criteria were included in the study if they met both of the following requirements: (a) aged 50–75 years; (b) CT images are available. The institutional review board of Cancer Hospital, Chinese Academy of Medical Sciences approved this retrospective study and waived the requirement for informed consent.

### Study group

In this study, we included noncalcified nodules ≥ 4 mm in diameter (nodule diameter refers to the maximum diameter) and defined these nodules as positive nodules. The correlation between nodule number and LC at the nodule and participant levels was explored. According to the needs of the research, the grouping criteria were developed through the integration of NCCN guidelines, Lung-RADS guidelines, and Chinese lung cancer screening guidelines (15–17). On the nodule level, the detected nodules were divided into three groups (diameter cut-off refer to maximum diameter): solid nodules (SNs)/solid components of partial SNs < 6 mm or non-SNs (NSNs) < 8 mm (Group A), SNs/solid components of partial SNs measuring 6-15 mm or NSNs measuring 8-15 mm (Group B), and SNs/solid components of partial SNs or NSNs ≥ 15 mm (Group C). On the participant level, the largest nodules of each participant were considered as the study object. The participants were divided into three groups based on the largest nodule: SNs/solid components of partial SNs < 6 mm or NSNs < 8 mm (Group Amax), SNs/solid components of partial SNs measuring 6-15 mm, or NSNs measuring 8-15 mm (Group Bmax), and SN/solid components of partial SNs or NSNs ≥ 15 mm (Group Cmax).

### LDCT scanning

All CT scans were obtained using 64-detector row scanners (LightSpeed VCT, Discovery CT750 HD or Optima CT660, General Electric Medical Systems; Healthineer or Edge, Siemens Medical Systems) at full inspiration. The CT scanning parameters were as follows: tube voltage, 120 kVp; automatic current time, 20–250 mA with rotation time of 0.5 s; and thickness, 5 mm. Reconstruction thicknesses were 1.0 and 1.25 mm, and the interval was 0.8 mm using a standard reconstruction algorithm. The “Dose Report” function of the spiral CT was turned on to record the dose parameters during the scan. The image data were transferred to the Picture Archiving and Communication System (PACS).

### CT imaging evaluation

Every baseline LDCT images were read by two chest radiologists (3-5 years of experience in thoracic radiology). All noncalcified nodules ≥ 4mm in diameter on LDCT images were identified, including intraparenchymal and endobronchial nodules. The number, size, and type of each nodule were recorded. LC nodules are confirmed by pathological examination, and a nodule is considered benign if it has not been confirmed as LC after the second round of screening. According to whether the nodule covered the lung parenchyma, the nodule was divided into the following: SN, part-solid nodule (PSN), or NSN. The number of nodules was recorded in strict accordance to the imaging record standard of the National Cancer Center. Each nodule was assigned a serial number requiring a detailed recording of its size (including the solid components size), shape, density, location, and distance from the pleura. The nodule number was defined as the number of noncalcified pulmonary nodules detected at the baseline screening. We defined five categories according to the number of nodules: one, two, three, four, and more than four nodules. If the two radiologists did not reach an agreement, a senior radiologist (at least 20 years of experience in thoracic radiology) makes the final decision.

### Statistical analyses

Normally distributed continuous variables are shown as mean ± standard deviation or 95% confidence interval (95%CI), whereas non-normally distributed continuous variables are presented as median (range). Categorical variables are shown as numbers (percentages). LC probability by nodule number groups for participants at baseline screening was evaluated by Chi-square test. In the stratified groups by participant and nodule risk levels, one-factor binary logistic regression analysis was performed to analyze the correlation between nodule number and LC probability. The Cochran–Armitage test for trend was conducted to assess LC trends over nodule number. All statistical data were analyzed using the Statistical Package for the Social Sciences version 26. The level of significance was defined as *p* < 0.05.

## Results

### LDCT detection results

**Figure 1:**
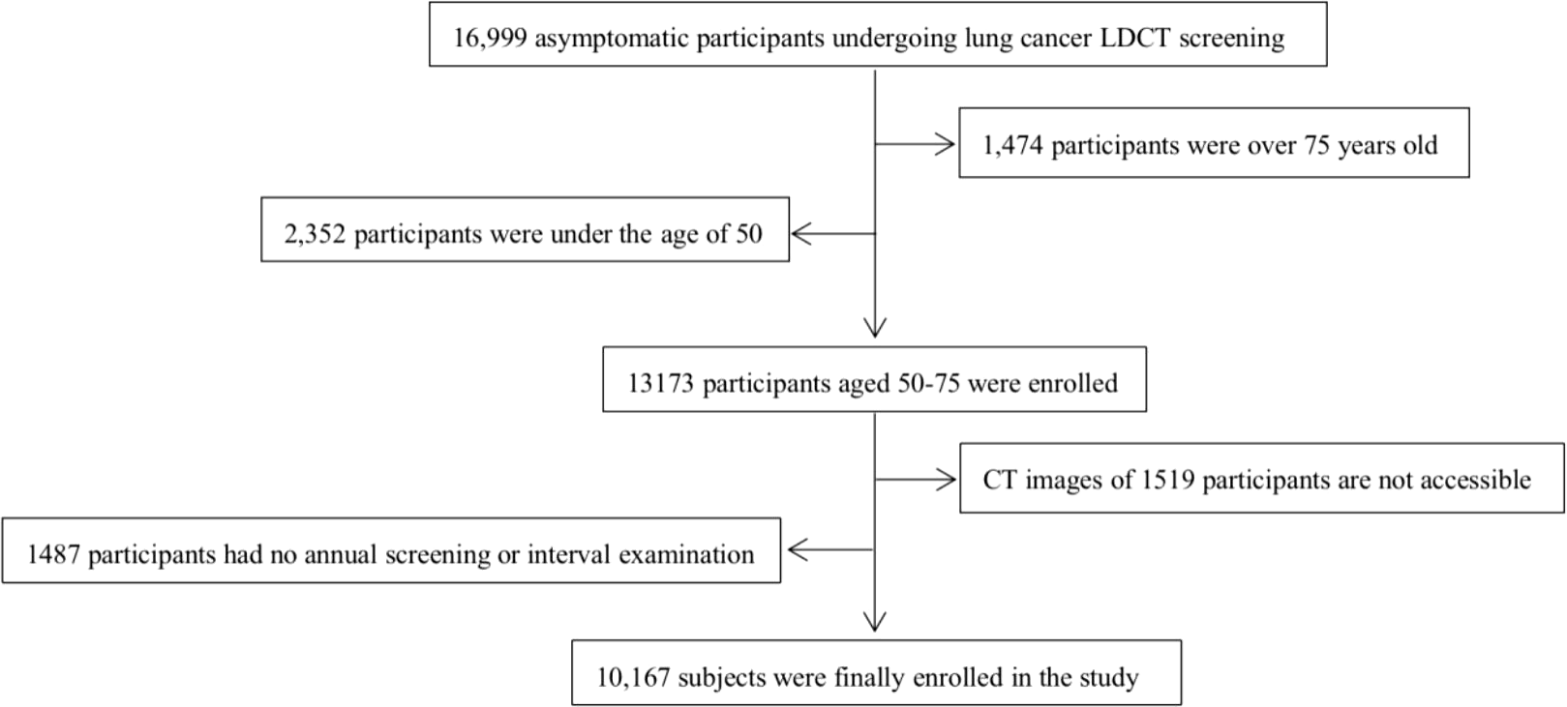
Flowchart of participants included in the analysis.

**Figure 2:**
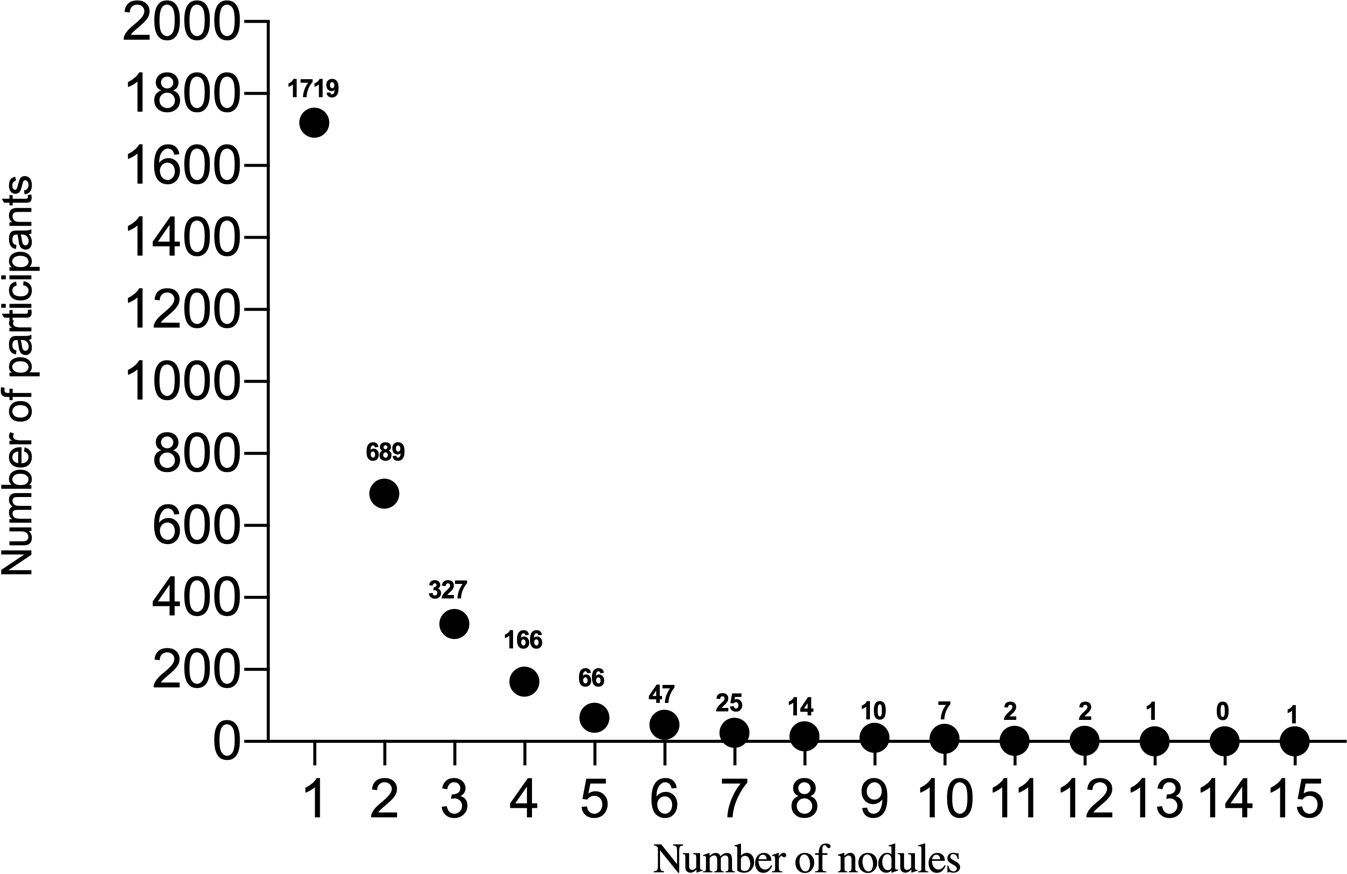
The horizontal axis represents the number of nodules, and the vertical axis represents the number of participants.

In this study, 10167 participants underwent LDCT examination including 4629 males and 5538 females, age range from 50-75 years (**Figure 1**). A total of 3076 participants with at least one non-calcified nodule were included in the study, 1486 males (48.3%) and 1590 females (51.7%), median age was 60 years old (IQR 57-63). The number of participants with noncalcified nodules decreased curvilinearly as the nodule numbers increased. The detailed distribution is shown in **Figure 2**. 1719 (55.9%), 689 (22.4%), 327 (10.6%), 166 (5.4%), and 175 (5.7%) participants had one, two, three, four, and more than four nodules, respectively. There were 1926 (62.6%), 1052 (34.2%), and 98 (3.2%) participants in the Amax, Bmax, and Cmax groups, respectively. In the Bmax group, with the increase in the nodule number, the proportion of participants increased from 25.2% to 63.4%. In the Amax group, with the increase in nodule numbers, the proportion of participants decreased from 72.1% to 29.1%. the proportion of participants in the Cmax group was also slightly increased (**Table 1**). In total, 5875 positive nodules were detected in 3076 participants, including 4611 SNs (78.5%), 437 PSNs (7.4%), and 827 NSNs (14.1%). As the number of nodules increased, the proportion of solid nodules increased from 76.5% to 80.6%. PSNs had the highest proportion in single nodules (8.9%), and NSNs had the highest proportion with > 4 nodules (15.3%) (**Table 2**). The largest nodules size increased linearly with more nodules detected (p  <  0.05).

**Table 1:** Characteristics of participants with at least one pulmonary nodule at baseline screening round.

		Number of nodules in participants				
		1	2	3	4	5
**Participants**						
**Sex**						
Male	1486	804	351	165	82	84
Female	1590	915	338	162	84	91
**Age**						
Median	60	60	60	60	60	60
IQR	57-63	58-63	56-63	56-62	58-63	58-63
**Group**						
Amax	1926	1239 (72.1)	404 (58.6)	158 (48.3)	74 (44.6)	51 (29.1)
Bmax	1052	433 (25.2)	268 (38.9)	156 (47.7)	84 (50.6)	111 (63.4)
Cmax	98	47 (2.7)	17 (2.5)	13 (4.0)	8 (4.8)	13 (7.5)

**Table 2:** Nodule characteristics detected at baseline screening round.

	Number of nodules in participants				
	1	2	3	4	5
**Nodule Size**					
Median	5	5	5	5	5
IQR	4-6	4-6	4-6	4-6	4-6
Nodule(Largest) Size*					
Median	5	6	6	6	7
IQR	4-6	5-7	5-7	5-8	6-9
**Nodule Type**					
Solid nodule	1315 (76.5)	1089 (79.0)	780 (79.5)	535 (80.6)	892 (78.7)
Part-solid nodule	153 (8.9)	96 (7.0)	72 (7.3)	48 (7.2)	68 (6.0)
Non-solid nodule	251 (14.6)	193 (14.0)	129 (13.2)	81 (12.2)	173 (15.3)
**Group**					
A	1239	1030	754	519	859
B	433	328	216	137	259
C	47	20	11	8	15

### Lung cancer detection

97 participants were pathologically confirmed to have LC, 37 (38.1%) males, median age was 61 years (IQR 58.5-64); 60 (61.9%) females, median age was 60 years (IQR 55-63). There were 87 people with stage I lung cancer, accounting for about 90.0%(87/97). 93, 90, 3, 2, 1, and 1 participant had non-small cell lung cancer, adenocarcinoma, squamous-cell carcinoma, small cell lung cancer, neuroendocrine carcinoma, and sarcoma, respectively. Simultaneous multiple tumors were found in 18 participants. In total, 15, 2, and 1 participant had two, three, and four tumors, respectively, all those multiple malignant tumors were pathologically confirmed to be adenocarcinoma. LC was confirmed with histopathology in the largest, second largest, and third largest nodules in 94/97 (96.9%), 2/97 (2.1%), and 1/97(1.0%) of the participants, respectively. The average nodule number was equal for both participants with and without LC (median nodule number, 1). The nodule-number ranges were 1–11 and 1–15 in participants with and without LC, respectively.

119 malignant nodules were confirmed in 97 participants with LC. Among the malignant nodules, 33 (27.7%), 37 (31.1%), and 49 (41.2%) were SNs, PSNs, and NSNs, respectively. The size of malignant nodules on thin-slice CT were 7–50 mm, and the median diameter was 12 mm.

### Relationship between nodule number and lung cancer probability

#### On the participant level

One nodule was found in 1719 participants, and 49 participants were diagnosed with LC (2.9%; 95% confidence interval [CI], 2.1–3.6%). Of the 689 participants with two nodules, 22 were diagnosed with LC (3.2%; 95% CI, 1.9–4.5%). Eleven of 327 participants with three nodules (3.4%; 95% CI, 1.4-5.3%), 6 of 166 with four nodules (3.6%; 95% CI, 0.7-6.5%) and 9 of 175 with more than four nodules (5.1%; 95% CI, 1.8-8.4%) were confirmed with LC. The nodule number was not associated with LC probability, although the LC probability slightly increased with the number of nodules. (**Table 3**) (p > 0.05).

**Table 3:** Lung cancer probability for participants at baseline screening.

Nodule count	Participants	Total Lung cancer	Lung cancer probability	95%CL	
				Low Bound	Upper Bound
1	1719	49	2.9%	2.1%	3.6%
2	689	22	3.2%	1.9%	4.5%
3	327	11	3.4%	1.4%	5.3%
4	166	6	3.6%	0.7%	6.5%
>4	175	9	5.1%	1.8%	8.4%

**Figure 3:**
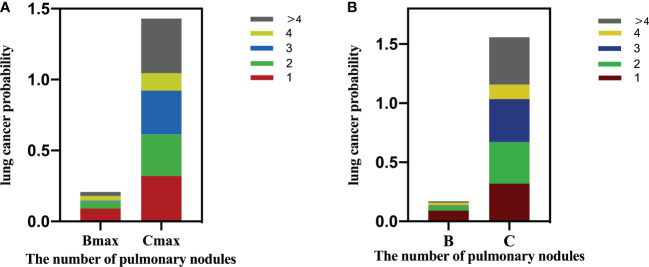
**(A)** Lung cancer probability by nodule count for Amax/Bmax/Cmax based on participant level. **(B)** Lung cancer probability by nodule count for A/B/C nodules based on nodule level.

When the participants were stratified according to their largest nodule (**Table 4**), in the Bmax groups, the probability of LC was significantly higher in participants with only one nodule than for those with >4 nodules (p < 0.05, **Figure 3**), and the probability of LC showed a negative linear trend with increased nodule numbers (p < 0.05). For the Cmax group, there was no difference in the probability of LC among participants with different numbers of nodules, although the probability of LC was increased to a certain extent (p > 0.05). In addition, we performed a subgroup analysis based on the female population. Only in the Bmax group, participants with isolated nodules had a higher probability of LC than participants with more than 4 nodules, with statistically significant differences (**Table S1**).

**Table 4:** Lung cancer probability by nodule count for Amax/Bmax/Cmax based on participant level.

		Amax		Bmax		Cmax	
Lung cancer		Yes	No	Yes	No	Yes	No
Nodule count	1	2	1237	39	394	15	32
	2	1	403	13	255	5	12
	3	2	156	1	155	4	9
	4	2	72	3	81	1	7
	>4	1	50	3	108	5	8
Total		8	1918	59	993	30	68

Group Amax: (SNs)/solid component of partial SNs < 6 mm or non-SNs (NSNs) < 8 mm

Group Bmax: SNs/solid component of partial SNs measuring 6-15 mm or NSNs measuring 8-15 mm

Group Cmax: SNs/solid component of partial SNs or NSNs ≥ 15 mm

*Statistical results (Group Bmax) are shown in Table S1

#### Based on nodule level

**Table 5** illustrates that as the nodule grade increased, the probability of LC also increased, displaying a positive linear trend (overall: Group A, 0.4%; Group B, 4.4%; and Group C, 32.7%; p < 0.01). For Group B nodules, the probability of malignancy was significantly higher when the number of nodules was 1 than those with more than four nodules (p < 0.01, **Figure 3**). However, for the nodules in groups A and C, the number of nodules had limited utility for distinguishing LC.

**Table 5:** Lung cancer probability by nodule count for A/B/C nodules based on nodule level.

		A		B		C	
Lung cancer*		Yes	No	Yes	No	Yes	No
Nodule count	1	2	1237	39	394	15	32
	2	1	807	13	308	7	13
	3	4	470	1	208	4	7
	4	4	292	3	131	1	7
	>4	1	306	3	232	6	9
Total		12	3112	59	1273	33	68

*We grouped all the nodules of the patients according to the standard, the number of lung cancer here refers to the number of cancer nodules, and the number of nodules refers to the total number of nodules in a patient.

Group A: (SNs)/solid component of partial SNs < 6 mm or non-SNs (NSNs) < 8 mm

Group B: SNs/solid component of partial SNs measuring 6-15 mm or NSNs measuring 8-15 mm

Group C: SNs/solid component of partial SNs or NSNs ≥ 15 mm

*Statistical results (Group B) are shown in Table S1

## Discussion

Many pulmonary nodules can be detected during LC screening, with most being benign. In this study, the detection rate of participants with positive nodules was 30.2% (3076/10167). The detection rates of LC in positive participants were 3.2% (97/3076) and approximately 0.95% (97/10167) in the entire population. The number of pulmonary nodules is frequently ignored during LC screening. A few studies suggest that the number of nodules does not differentiate benign from malignant nodules, but more in-depth research is still required (18, 19). At present, only a few risk-prediction models have considered the number of nodules as a risk factor (20, 21). This study innovatively stratified nodules according to the nodule size and determined the correlation between the nodule number and the probability of LC. Our principal findings include the following. First, in 55.9% (1719/3076) of the participants, a solitary nodule was detected at baseline. Second, the size of the largest nodule increased with the number of detected nodules increasing, and this trend was linear. At present, the risk of LC is often evaluated according to the largest nodule (22). When the largest nodule diameter increases with the number of detected nodules increasing, this may indicate an indirect association between nodule number and LC incidence. Third, we also found that not all LC nodules detected in this study were the largest nodules (94/97), but the largest nodules had the highest possibility of malignancy (Group C, 32.7%). Fourth, generally, although the LC probability slightly increased with the number of nodules, there is no statistically significant difference. Fifth, when stratified by nodule size, the effect of nodule number on LC probability was nodule-size dependent. On the participant level, in the Bmax group, the probability of LC was significantly higher in participants with only one nodule than in participants with >4 nodules, and the number of nodules showed a negative linear trend with the probability of LC. On the nodule level, in the Group B, the probability of LC in participants with only one nodule was significantly higher than those with >4 nodules. For nodules in Group A or C, the number of nodules had a limited association on LC probability at both the participant and nodule levels.

Multiple noncalcified nodules in Group A were usually < 6 mm in diameter. Small nodules in this size range are frequently observed in routine clinical practice and are usually benign (23). In our study, only 8 of 1926 people were confirmed with LC. They typically reflect previous infection or healing granulomas of lymph nodes in the lungs. For multiple noncalcified nodules with at least one Group B nodule (SNs/solid component of PSNs measuring 6-15 mm or NSNs measuring 8-15 mm), follow-up at approximately 3-6 months is recommended. Our findings demonstrate that the risk of primary cancer decreased as the total number of nodules increased from 1 to 4. When participants had only one nodule, LC probability was significantly higher than when participants had >4 nodules. In participants with multiple nodules with at least one nodule that was 15 mm or larger (Group C nodules), infectious causes or multiple primary adenocarcinomas are considered (24). In this case, our results indicated that the number of nodules has limited utility for the prediction of LC. There are two possible explanations. First, it may be due to insufficient sample size, as the number of nodules detected in Group C was much less than the other two groups. Second, the nodule diameter of the Cmax group was > 15 mm (categorized regularly as high-risk nodule grade classification). This may contribute to the limited effect of the number of nodules on the probability of LC.

The average nodule number of each screening participant was significantly lower in the present study than in McWilliams et al.’s study (25). The participants without LC in the British Columbia Cancer Agency (BCCA) study had an average of 10 nodules, and the participants without LC in the Pan-Canadian Early Detection of Lung Cancer Study (PanCan) study had an average of 6.2 nodules. The average nodule number of participants without LC in our study was 1.9. The average numbers of participants with LC in the BCCA study, PanCan study, and our study were 4.7, 4.8, and 2.1, respectively. The above differences can be explained by the different inclusion criteria. A risk prediction model determined the inclusion criteria in the PanCan study, and the risk of LC in the participants included in the study for 3 years was at least 2%. The participants included in the BCCA study had at least 30 years of current or previous smoking history. The present study was an opportunistic screening study with no specific requirements for smoking history, and the overall LC risk of the study participants in this study was lower than those of the BCCA and PanCan studies. Moreover, all noncalcified nodules larger than 1 mm in diameter were included in PanCan and BCCA study, and we included noncalcified nodules ≥ 4 mm in diameter. A smaller threshold will undoubtedly increase the number of positive nodules detected.

Although a considerable amount of time is spent on numbering all nodules (≥ 4 mm in diameter), this is necessary for screening. First, smaller nodules may still grow during follow-up, and the changes between the two scans will affect follow-up and clinical decisions. Moreover, new nodules are regularly detected in annual screening and carry a higher LC probability than baseline nodules, even for smaller sizes (9, 26). However, some studies have also reported that new subsolid nodules are associated with a lower incidence of LC and a higher spontaneous regression rate, indicating that they have greater inflammatory potential (27, 28). Although it remains debatable, considering the potential risk of LC, it is necessary to record baseline nodules in detail as a reference.

This study had some limitations. First, this study included all noncalcified nodules and did not differentiate SNs, PSNs, and NSNs. The influence of nodule numbers from different types of nodules (solid, subsolid) on the probability of LC should be further studied in detail. Second, participants’ smoking status were not included in this study because this part of the data was not available for some reasons. Since opportunistic screening does not restrict for smoking levels, it does not affect the experimental results and we will refine it in future trials. Finally, this was a single-center study, and multicenter studies are required to further verify our results.

## Conclusions

In LDCT lung cancer screening, lung cancer probability does not significantly change with the number of nodules. However, assessing the nodule number in conjunction with nodule size in baseline LDCT lung cancer screening is considered beneficial. More attention should be paid to and active follow-up should be provided for solitary nodules, especially SNs/solid component of PSNs measuring 6–15 mm or NSNs measuring 8–15 mm.

## Data availability statement

The raw data supporting the conclusions of this article will be made available by the authors, without undue reservation.

## Ethics statement

The institutional review board of Cancer Hospital, Chinese Academy of Medical Sciences approved this retrospective study and waived the requirement for informed consent.

## Author contributions

Conceptualization: QW, NW. Methodology: QW, SZ, NW. Methodology validation: SZ, NW. Investigation: QW, SZ, LQ, LZ. Data Curation: ZZ, JZ, HL. Formal analysis:QW, YX, LQ. Writing - Original Draft: QW. Writing - Review and Editing: YH, JW, WT, NW. Visualization: YX, QW. Supervision: SZ, NW. Funding acquisition: NW. All authors contributed to the article and approved the submitted version.
