# Supplementary material for: Correlation between lung cancer probability and number of pulmonary nodules in baseline computed tomography lung cancer screening: A retrospective study based on the Chinese population
Source: Front Oncol. 2023 Jan 4;12:1061242. doi: 10.3389/fonc.2022.1061242 (PMC9846312; doi:10.3389/fonc.2022.1061242)
Supplement: Supplementary file 1 [file Table_1.docx]

**Table S1**

**Table S1 Lung cancer probability by nodule count for Amax/Bmax/Cmax based on female subgroup**

|  | | **Amax** | | **Bmax** | | **Cmax** | |
| --- | --- | --- | --- | --- | --- | --- | --- |
| Lung cancer | | Yes | No | Yes | No | Yes | No |
| Nodule  count | 1 | 0 | 663 | 29 | 194 | 10 | 19 |
|  | 2 | 0 | 211 | 10 | 110 | 1 | 6 |
|  | 3 | 1 | 84 | 1 | 70 | 1 | 5 |
|  | 4 | 1 | 35 | 2 | 40 | 1 | 5 |
|  | ＞4 | 0 | 26 | 2 | 57 | 1 | 5 |
| Total | | 2 | 1019 | 44 | 471 | 14 | 40 |
| Group Amax: (SNs)/solid component of partial SNs < 6 mm or non-SNs (NSNs) < 8 mm  Group Bmax: SNs/solid component of partial SNs measuring 6-15 mm or NSNs measuring 8-15 mm  Group Cmax: SNs/solid component of partial SNs or NSNs ≥ 15 mm | | | | | | | |
